# Organic Electrochemical Neurons: Nonlinear Tools for Complex Dynamics

**DOI:** 10.1021/acsaelm.5c01710

**Published:** 2025-11-20

**Authors:** Gonzalo Rivera-Sierra, Roberto Fenollosa, Juan Bisquert

**Affiliations:** Universitat Politècnica de València-Consejo Superior de Investigaciones Científicas, 83167 Instituto de Tecnología Química, Camino de Vera, València 46022, Spain

**Keywords:** organic electrochemical transistor (OECT), nonlinear dynamics, Hopf bifurcation, neuromorphic electronics, bioinspired circuits, organic mixed ionic–electronic conductors (OMIEC)

## Abstract

Hybrid oscillator architectures that combine feedback oscillators with self-sustained negative resistance oscillators have emerged as promising platforms for artificial neuron design. In this work, we introduce a modeling and analysis framework for amplifier-assisted organic electrochemical neurons leveraging nonlinear dynamical systems theory. By formulating the system as coupled differential equations describing membrane voltage and internal state variables, we identify the conditions for self-sustained oscillations and characterize the resulting dynamics through nullclines, phase space, and bifurcation analysis, providing complementary insight into standard circuit-theoretic arguments of the operation of oscillators. Our simplified yet rigorous model enables tractable analysis of circuits integrating classical feedback components (e.g., operational amplifiers) with devices exhibiting negative differential resistance such as organic electrochemical transistors (OECTs). This approach reveals the core mechanisms behind oscillation generation, demonstrating the utility of dynamic systems theory in understanding and designing complex hybrid circuits. Beyond neuromorphic and bioelectronic applications, the proposed framework offers a generalizable foundation for developing tunable biologically inspired oscillatory systems for sensing, signal processing, and adaptive control.

## Graphical abstract

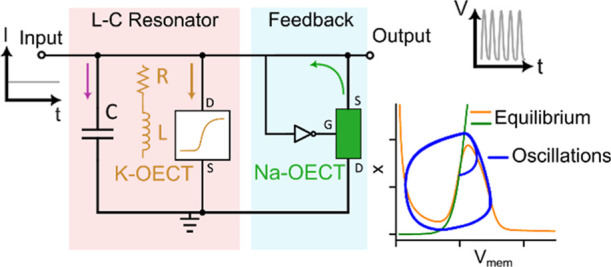

## 1 Introduction

Emulating the spiking behavior of biological neurons using organic electronics has emerged as a promising avenue for neuromorphic computing and biointerfacing applications. Among the various technologies explored, organic electrochemical transistors (OECTs) based on organic mixed ionic–electronic conductors (OMIECs)^1–3^ have attracted significant interest due to their intrinsic properties that enable complex signal processing and biocompatibility. The integration of OECTs as the foundational element in biologically inspired spiking oscillators represents a significant and promising strategy for emulating the functional behavior of neurons.^4–12^ These devices play a central role in enabling perception-responsive systems that operate at the interface of ionic and electronic signaling, closely mimicking the way biological neurons process and respond to stimuli, and using similar physical principles to generate biocompatible oscillations.^11,13^

A critical feature for achieving spiking dynamics in OECTs is a regime of negative transconductance, either through antiambipolar behavior, resulting from partially stacked p–n heterojunctions in the transistor channel,^6^ or through saturation effects in the density of states.^14^ In previously reported architectures, OECTs have been configured into networks of multiple transistors coupled with feedback-driven amplification stages, to replicate essential features of neural excitability and spiking.^6^ However, the complexity of such multicomponent systems poses challenges in integration, stability, and modeling.

In parallel, traditional oscillator theory has long provided analytical tools for understanding self-sustained oscillations in classical feedback-based circuits.^15–17^ These include criteria such as those of Barkhausen, Nyquist, and Routh-Hurwitz, which analyze the loop gain, phase conditions and characteristic equations of linearized systems to determine the onset of oscillations. Complementary, time-domain analyses focus on the behavior of the damping terms and the system’s response to perturbations. These classical approaches are robust but rely heavily on the formulation of transfer functions.^18–21^

More recently, attention has also shifted toward oscillators built from devices with emergent nonlinear physical properties, such as those exhibiting negative differential resistance. For example, vanadium dioxide (VO_2_) devices that undergo an insulator–metal transition have been used to realize self-oscillators: when biased into the negative differential resistance region, Joule heating triggers a phase-change cycle that produces sustained voltage oscillations without the need for a conventional resonator.^22^ Other platforms include devices based on NbO_2_ or chalcogenides with S-shaped I–V curves that support relaxation-type oscillations.^23^ In parallel, spintronic nanooscillators (STNOs) have emerged as another class of compact, energy-efficient self-sustained oscillators. These devices leverage magnetization dynamics driven by spin-transfer or spin–orbit torques to generate microwave-frequency oscillations.^24^

Building on these emerging devices, oscillatory neural networks (ONNs) based on self-sustained oscillators have recently emerged as a powerful alternative to conventional neuromorphic systems. By encoding information in the relative phases of coupled oscillators rather than through discrete spikes or voltages, ONNs enable highly parallel, energy-efficient and dynamically reconfigurable computation—offering significant advantages for real-time and low-power applications.^25^

In these systems, the interplay between the device’s intrinsic relaxation dynamics and circuit-level capacitive elements forms an effective resonant system. Self-sustained oscillations^26^ are maintained not by traditional feedback loops but rather by the device’s own nonlinear response—representing a fundamentally different mechanism for achieving oscillatory behavior.^27,28^ In this context, the analysis of artificial neurons can be undertaken from widely used computational neuroscience models that stem from the Hodgkin-Huxley model for the squid axon^29,30^ and provide major tools to understand action potential dynamics and spiking.^31–33^ These models rely on the framework of nonlinear dynamical systems, which are governed by differential equations involving time-dependent variables and fixed parameters.^27,34,35^ Variables represent quantities such as internal voltage *u* or internal conductance that evolve over time, while parameters like capacitance or input current *I*_0_ remain constant during operation.

In the context of artificial spiking neurons, including those based on organic electrochemical systems, the Hopf bifurcation provides a theoretical framework to explain how rhythmic spiking activity emerges from the interplay between system nonlinearity and feedback.^27^

Bridging these two worlds, feedback oscillators and self-sustained negative resistance oscillators, hybrid oscillator architectures have emerged as a compelling research direction. These systems combine feedback elements (e.g., operational amplifiers) with nonlinear devices like OMIEC-based OECTs, enabling the construction of compact, highly tunable, and biologically inspired and compatible spiking circuits.^6^ Despite their potential, however, a comprehensive theoretical framework that captures the behavior of such hybrid systems, rooted in nonlinear dynamics rather than purely circuit-theoretic arguments, is still lacking.

In this work, we aim to fill that gap. We present a modeling and analysis framework for amplifier-assisted organic electrochemical neurons using tools from nonlinear dynamical systems theory. By formulating the system as a set of coupled differential equations involving membrane voltage and internal state variables, we identify the conditions under which self-sustained oscillations emerge and characterize them in terms of nullclines, phase-space trajectories, and bifurcation analysis.

In this way, it becomes possible to directly design and control the types of oscillations required for a specific application. While this work focuses on neuromorphic applications, particularly neuron-like behavior, the methodology can be readily extended to other domains, as it is grounded in general oscillator circuit principles. The universality of the underlying dynamics makes the proposed approach versatile and applicable across a broad range of technologies that rely on oscillatory behavior.^36,37^

## 2 Results and Discussion

### 2.1 Hopf Bifurcation Characteristics

Before analyzing the specific hybrid OECT–amplifier oscillator, it is useful to establish the general theoretical framework used to characterize the onset of self-sustained oscillations in nonlinear dynamical systems. Here, we briefly outline the Hopf bifurcation mechanism, which provides the mathematical foundation for understanding how a stable equilibrium loses stability and gives rise to a limit-cycle oscillation. This general analysis, based on the linearization of the system around its steady state and evaluation of the Jacobian trace and determinant, forms the basis for all subsequent modeling and simulations presented in this work. By introducing these concepts first, we ensure that the dynamic features observed later in the hybrid OECT system can be interpreted in terms of well-defined stability criteria and bifurcation behavior.

A basic dynamical model is formed by coupled differential nonlinear equations

1 $$\frac{du}{dt}=F\left(u,x,I_{0}\right)$$

2 $$\frac{dx}{dt}=G\left(u,x\right)$$

here *x* is an internal state variable and *F* and *G* are multivalued nonlinear functions. The nullclines represent the set of points in the system where a particular variable does not change over time, meaning its rate of change is zero. They are defined by the conditions

3 $$F\left(u,x,I_{0}\right)=0$$

4 G(u,x)=0

Dynamical systems theory^38,39^ provides essential tools for understanding the long-term behavior of nonlinear systems such as eqs 1, 2 by analyzing the asymptotic trajectories in phase space, which represent the evolution of system variables over time. These trajectories can converge to fixed points, diverge, or form closed loops known as limit-cycles—that correspond to sustained oscillations. By studying the stability of steady states, one can predict whether the system will remain at rest or transition to an active oscillatory regime.

Crucially, even small changes in system parameters—such as ionic conductivity, gate voltage, or capacitance—can trigger qualitative changes in behavior. The central mechanism underlying the sensitivity to the occurrence of oscillations is the Hopf bifurcation, a phenomenon where a stable fixed point loses stability as a parameter crosses a critical threshold, giving rise to a limit-cycle. This marks the onset of self-sustained oscillations from a previously quiescent state.

To evaluate the stability of the equilibrium points of the system, which correspond to the intersections between the nullclines *F*(*u*,*x*) = 0 and *G*(*u*,*x*) = 0 of eqs 3, 4, we analyze the local linearization of the dynamical equations around a given fixed point. This approach allows us to determine whether the operating regime is stable or oscillatory, following the standard procedure of bifurcation analysis.

By applying small perturbations Δ*u* and Δ*x* to the steady-state values (*u*_eq_, *x*_eq_), eqs 1, 2 can be linearized as

5 $$\frac{d}{dt}\left[\begin{array}{l} \Delta u\\ \Delta x \end{array}\right]=J_{X}\left[\begin{array}{l} \Delta u\\ \Delta x \end{array}\right]$$

where *J*_*x*_ is the Jacobian matrix of the system evaluated at the equilibrium point

6 $$J_{X}={\left[\begin{array}{ll} F_{u} & F_{x}\\ G_{u} & G_{x} \end{array}\right]}_{u_{eq},x_{eq}}$$

with *F*_*u*_ = ∂*F*/∂*u*, *G*_*u*_ = ∂*G*/∂*u*, *F*_*x*_ = ∂*F*/∂*x*, and *G*_*x*_ = ∂*G*/∂*x*.

Then, the trace and determinant of the Jacobian

7 $$T_{\lambda }=F_{u}+G_{x}$$

8 $$\Delta_{\lambda }=F_{u}G_{x}-F_{x}G_{u}$$

provide a compact description of the system’s local stability and allow us to classify the different dynamical regimes. When the determinant is positive Δ_λ_ > 0 and the trace is negative *T*_λ_ < 0, the equilibrium point is locally attractive, leading the system to a stable, non-oscillatory state where any perturbation decays back to equilibrium. Conversely, when both the determinant and the trace are positive Δ_λ_ > 0, *T*_λ_ > 0, the fixed point becomes repulsive and trajectories spiral outward, giving rise to a self-sustained limit-cycle that defines the oscillatory regime. The boundary between these two behaviors occurs when the trace crosses zero *T*_λ_ = 0 while the determinant remains positive, corresponding to the onset of a Hopf-type bifurcation. At this critical condition, the system exhibits small-amplitude sinusoidal oscillations with an angular frequency given by $\omega_{0}\approx \sqrt{\Delta_{\lambda }}$ .

### 2.2 Model Description

Following the experimental demonstration of a hybrid OECT oscillator by Harikesh et al.,^6^ we use that architecture as a reference framework to construct a minimal theoretical model. This model allows us to reproduce and analyze, through numerical simulations, the key dynamic features observed in the experiments. The schematic of the oscillator circuit, originally reported by Harikesh et al., is shown in Figure 1a, where it was interfaced with a biological mouse model. As indicated in Figure 1a, the neuronal analogy is inspired by the combination of ion channels in the Hodgkin–Huxley model,^29,30^ including both a potassium and a sodium channel, where the latter contains a negative transconductance feature that enables the sustained oscillations. In this setup, the system demonstrates a reproducible physiological response through the generation of autonomous spiking activity within a biorelevant frequency range, all achieved with low operating voltages on the order of millivolts, Figure 1b. This experimental result confirms the broader principle discussed above: the oscillator not only emulates key neuronal functions, but also interacts directly with living organisms, effectively integrating neuromorphic electronics with biological systems at the interface of bioelectronics and biophysics.

**Figure 1:**
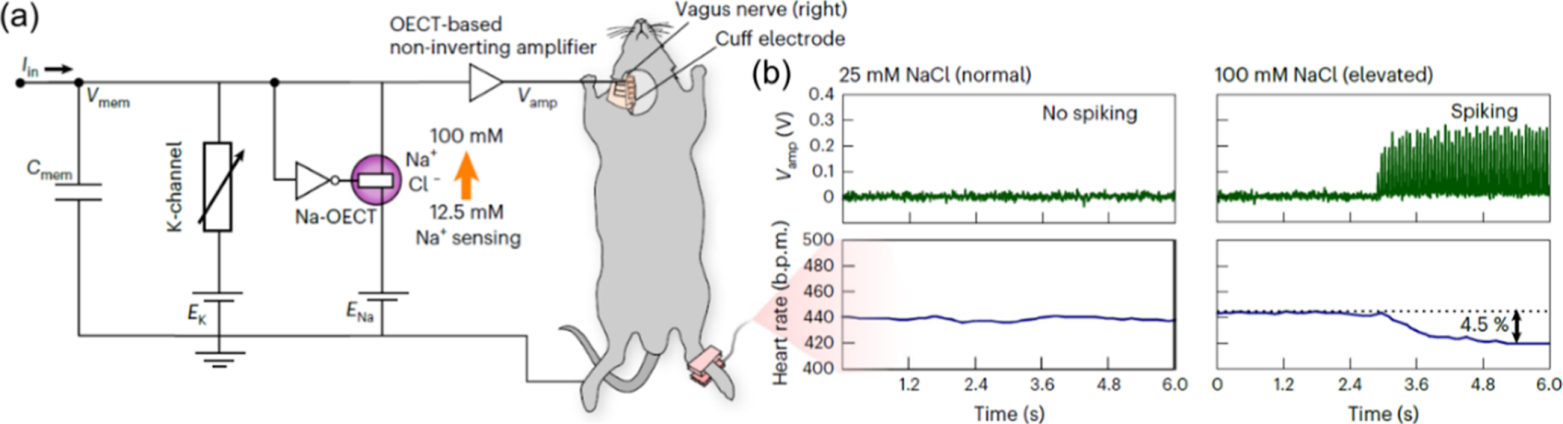
(a) OECT-based circuit showing sensing of Na + ions at the Na-OECT and integration with the vagus nerve using an OECT-based amplifier and cuff electrodes. (b) Amplifier outputs at low (25 mM) and high (100 mM) concentrations of NaCl and the corresponding heart rate variation. The black horizontal dashed line represents the baseline heart rate. The purpose of this demonstration is to show the potential of OECT-based oscillators to sense biochemical signals and interface with nerves and does not imply that new therapeutic means are developed. Reproduced from Harikesh, P. C. et al., Nat. Mater. 2023, 22, 242–248, licensed under a Creative Commons Attribution 4.0 International License (CC BY 4.0).^6^

The implications for biophysical applications are extensive. However, in order to fully exploit the potential of such systems, both for biological integration and for high-performance and scalable technological platforms, it is essential to understand the underlying circuit theory and the precise mechanism by which these oscillations are generated. Thus, in the following, we develop a nonlinear dynamical approach that, to our knowledge, has not been shown previously.

We analyze the differential equations governing the system of Figure 1a by constructing an equivalent circuit (Figure 2) and employing transistor models that accurately capture the relevant physical behavior. As a first simplification, we describe the potassium transistor branch by assuming a constant applied potential *E*_K_ and neglecting any significant modulation of its current–voltage characteristics due to variations in the drain–source voltage (*V*_DS_) during oscillations. This assumption does not imply that *V*_DS_ itself is small, but rather that the variations of it within the selected operating range have only a minor influence on the transistor’s steady-state response. In practice, this condition is also desirable experimentally since achieving reproducible oscillations requires operating the device in a regime where the drain–source modulation does not substantially distort the channel conductance. Under these circumstances, the potassium branch can be effectively represented as a resistive channel with a sigmoidal activation profile and a characteristic relaxation time

9 $$I_{K}=g_{K}xV_{mem}$$

10 $$x_{eq}\left(V_{mem}\right)=\frac{1}{1+e^{-\left(V_{mem}-V_{x}\right)/V_{x}}}$$

11 $$\tau_{K}\frac{dx}{dt}=x_{eq}-x$$

where *x*_eq_ represents the steady-state value of the variable *x*, τ_*K*_ denotes the relaxation time associated with the activation of the channel, and *V*_*K*_ and *V*_*m*_ correspond to the onset voltage and the steepness factor of the sigmoidal function, respectively.

**Figure 2:**
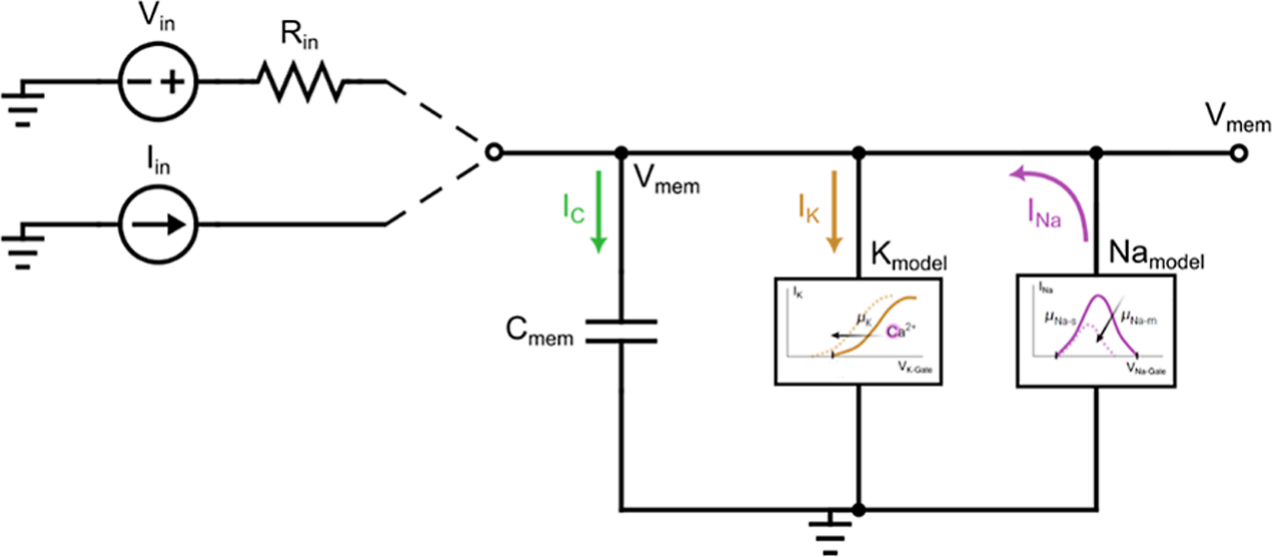
Equivalent circuit schematic of the OECT-based oscillator, inspired by the configuration shown in Figure 1. It is assumed a quasi-static regime where the transfer function remains invariant with respect to the low evolution of the membrane potential *V*_mem_. The sodium ionic current channel contributes a feedback path to the rest of the circuit. The circuit can be operated either under a constant current input or using a constant voltage source with an input series resistor. Graphs inside boxed panels reproduced from Harikesh, P. C. et al., Nat. Mater. 2023, 22, 242–248, licensed under a Creative Commons Attribution 4.0 International License (CC BY 4.0).^6^

Here, the parameters *g*_*K*_, *V*_*m*_, *V*_*K*_, and τ_*K*_ vary depending on the experimental characteristics of the transistor and the applied activation voltage *E*_*K*_. These must be properly adjusted to match the actual device response and reproduce the correct dynamic behavior in the model.

Now, turning to the analysis of the sodium branch, the first simplification arises from describing the voltage applied to the gate of the transistor as a function of the membrane voltage presented in the circuit. To do this, we implement the simplest possible form of an inverting amplifier: a resistive network *R*_in_ and *R*_f_ connected to the inverting input of an operational amplifier, while a baseline voltage *V*_0_ is applied to the noninverting input. By feeding the membrane voltage *V*_mem_ into the resistive input network and connecting the amplifier output *V*_G_ to the gate of the sodium transistor, we realize a complete inverting amplifier configuration, as shown in Figure 3.

**Figure 3:**
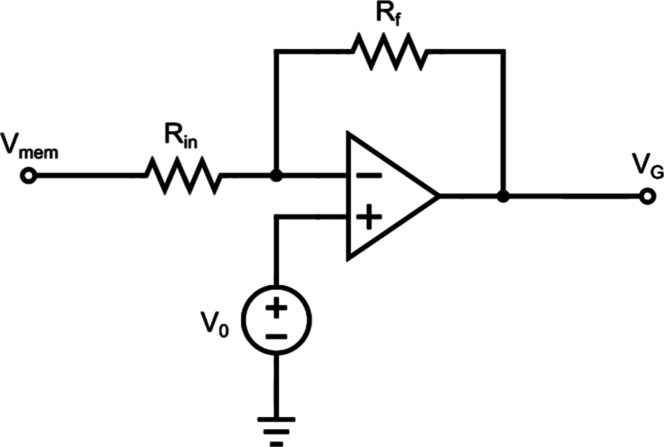
Schematic of the inverting amplification stage implemented with an operational amplifier and a resistive feedback network. The membrane voltage *V*_mem_ is applied at the input of the resistive divider, while the output of the operational amplifier provides an inverted and amplified signal that drives the gate terminal of the Na-OECT.

The relationship between the gate voltage and the membrane voltage is given by the amplifier gain, *A*, which depends on the resistance values in the feedback network.

12 $$A=1+\frac{R_{f}}{R_{in}}$$

13 $$V_{G}={AV}_{0}-\left(\frac{R_{f}}{R_{in}}\right)V_{mem}$$

In this configuration, the operational amplifier plays a central role in shaping the behavior of the hybrid oscillator. Its inversion function is essential to properly bias the sodium transistor in reverse, allowing the current through this branch to flow back into the circuit and provide the required negative feedback to sustain oscillations. Beyond this basic function, the amplifier also enables the precise alignment of the operating voltage ranges of the sodium and potassium branches. By the adjustment of the amplifier gain and offset, the current–voltage response of the sodium transistor can be shifted so that its region of negative transconductance overlaps with the voltage window in which the potassium branch exhibits its resistive transition. This matching is a necessary condition for periodic operation, as both devices must respond within the same potential range to support charge exchange and regenerative feedback. Furthermore, the amplifier isolates the sodium transistor from large drain–source voltage variations, ensuring that its conductive response remains effectively stationary throughout each oscillation cycle, which is a prerequisite for achieving reproducible oscillations. Finally, the feedback parameters introduced by the amplifier, such as the gain ratio *R*_f_/*R*_in_, provide an additional external and tunable degree of freedom that allows control over the oscillation frequency and the equilibrium bias point of the system.

Considering this configuration, the current flowing through the sodium branch, in the direction shown in Figure 2, can be described as

14 $$I_{Na}=g_{Na}n\left(V_{G}\right)V_{G}=g_{Na}n\left(V_{G}\right)AV_{0}-\frac{R_{f}}{R_{in}}g_{Na}n\left(V_{G}\right)V_{mem}$$

where *n*(*V*_*G*_) represents an internal variable that performs the activation and deactivation of the sodium channel. We describe this variable as a function of *V*_*G*_ for simplicity and because of its direct relationship with the transistor’s characteristic current–voltage curve. However, as previously discussed, this gate voltage is directly dependent on the membrane voltage. This *n*(*V*_*G*_) function could be modeled either as a Gaussian function with a maximum of 1 or as the difference of two sigmoid functions, similar to the one described in eq 10, but with shifted threshold values. Accordingly, the internal activation variable of the sodium transistor was modeled as the difference between two shifted activation functions, as mentioned before: the positive branch corresponding to activation and the negative one to deactivation, following the expressions

15 $$n\left(V_{G}\right)=a\left(V_{G}\right)-d\left(V_{G}\right)$$

$$a\left(V_{G}\right)=\frac{1}{1+e^{-\left(V_{G}-V_{a}\right)/V_{m_{a}}}},\left(activation\right)$$ 16

$$d\left(V_{G}\right)=\frac{1}{1+e^{-\left(V_{G}-V_{d}\right)/V_{m_{d}}}},\left(deactivation\right)$$ 17

where *V*_*a*_ and *V*_*d*_ are the activation and deactivation, respectively, threshold voltages of the sodium channel, while *V*_ma_ and *V*_md_ are the corresponding steepness factors leading the transition slopes. Depending on the specific experimental characteristics of the sodium transistor under study, both *n*(*V*_*G*_) and *g*_Na_ can be tuned to accurately reproduce the transistor’s response to gate voltage *V*_*G*_.

In this analysis, we assume that the sodium channel activation voltage *E*_Na_ is chosen such that the voltage difference between the drain and source does not vary significantly within the membrane voltage range relevant to the oscillatory regime. If this condition is not met, then it becomes necessary to explicitly model the dependence of *g*_Na_ and *n* based on the drain-source voltage difference (*V*_DS_ = *E*_Na_–*V*_mem_).

Additionally, we consider the activation and deactivation of the sodium channel to be instantaneous, based on the assumption that its dynamics are significantly faster than that of the potassium channel. This approximation allows us to simplify the model by avoiding an additional dynamic equation, while still capturing the essential behavior of the system. A more detailed analysis that relates this assumption, by explicitly introducing a third dynamic variable for the sodium channel, is presented in the final part of the next Section, where it is shown that the resulting three-variable model reproduces the same oscillatory behavior as the simplified two-variable formulation.

### 2.3 Simulations and Analysis

The starting point for analyzing the dynamic behavior of the hybrid OECT oscillator is the steady-state current–voltage characteristics of the individual transistor branches, which define the nonlinear elements of the system. These responses are presented in Figure 4, where panels (a) and (b) show the simulated current–voltage characteristics of the potassium and sodium OECT models, respectively.

**Figure 4:**
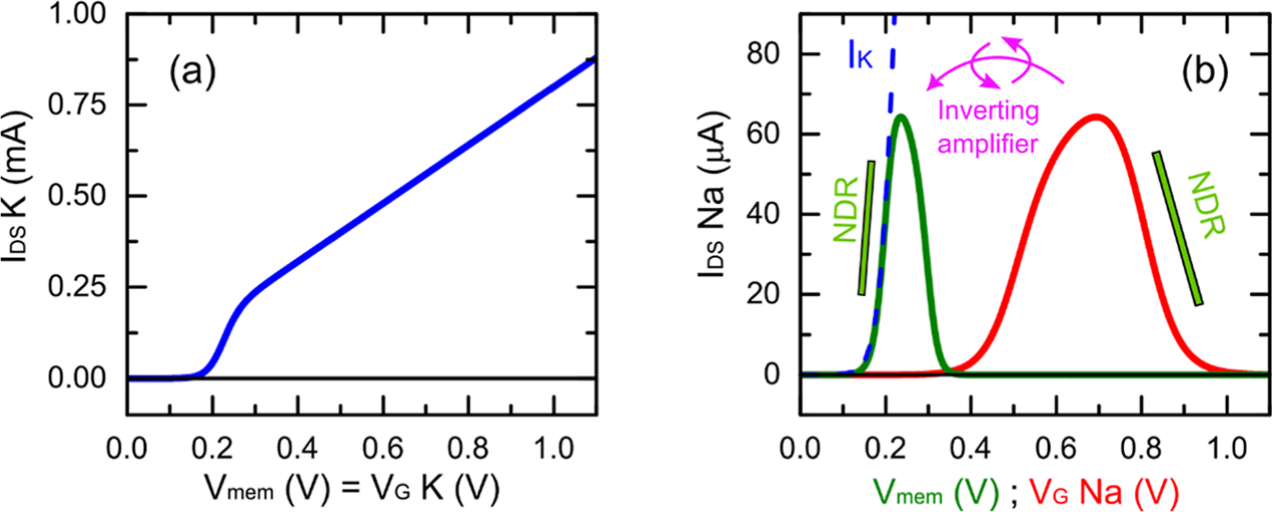
Simulated steady-state current–voltage characteristics of the OECT models used in this work. (a) Potassium transistor current as a function of the membrane potential, which in the model corresponds to the gate voltage. (b) Sodium transistor current plotted as a function of the gate voltage (red) and as a function of the membrane potential after the action of the inverting amplifier (green). The dashed blue line shows the potassium transistor current for comparison, illustrating that, due to the amplifier inversion, both devices now operate within the same voltage range but with opposite current directions. The parameters employed in the simulations are *g*_*K*_ = 0.80 mS; *V*_*x*_ = 0.22 V; *V*_*mx*_ = 0.02 V; *R*_f_ = 600 *k*Ω; *R*_in_ = 200 *k*Ω; *V*_0_ = 0.35 V; *g*_Na_ = 0.10 mS; *V*_*a*_ = 0.50 V; *V*_ma_ = 0.04 V; *V*_*d*_ = 0.80 V; *V*_md_ = 0.04 V.

As observed in Figure 4a, the potassium transistor exhibits a typical activation-type behavior with its channel conductance increasing monotonically once the gate potential exceeds the activation threshold. In contrast, the sodium transistor (red curve in Figure 4b) is activated at substantially higher voltages, well outside the operating range of the potassium branch. When the inverting amplifier is introduced, as discussed in the previous section, the sodium branch is effectively biased in reverse, inverting its current response and shifting its operating window to lower voltages (green curve in Figure 4b).

Due to this inversion, the activation of both branches can now be aligned within the same voltage range, as shown by the overlap between the solid green and dashed blue curves in Figure 4b, but with opposite current directions. In this configuration, the sodium branch behaves as a negative-resistance element relative to the potassium branch, thus providing the regenerative feedback required to sustain oscillations in the hybrid circuit.

With these nonlinear steady-state characteristics established, we can now proceed to perform a nonlinear dynamic analysis of the full oscillator circuit. The combination of the capacitor, the potassium and sodium branches, and the inverting amplifier forms a feedback network whose state variables evolve according to a set of coupled differential equations. These equations describe the time evolution of the membrane potential and the internal activation variable from which the phase-space trajectories and bifurcation behavior of the system can be derived.

To capture this dynamic interaction quantitatively, we formulate the current balance equation at the membrane node, which includes the membrane capacitive term *C*_mem_, the current flowing through each transistor branch, and the contribution of the inverting amplifier. This yields the following expression for the total input current

18 $$I_{in}=C_{mem}\frac{dV_{mem}}{dt}+g_{K}xV_{mem}-g_{Na}n\left(V_{G}\right)V_{0}+\frac{R_{f}}{R_{in}}g_{Na}n\left(V_{G}\right)V_{mem}$$

Combining this equation and eq 10, we can now derive the two differential equations, *F*(*V*_mem_,*x*) and *G*(*V*_mem_,*x*), that govern the dynamics of the system in terms of the two state variables

19 $$F=\frac{dV_{mem}}{dt}=\frac{1}{C_{mem}}\left[I_{in}-g_{K}xV_{mem}+g_{Na}n\left(V_{G}\right)V_{0}-\frac{R_{f}}{R_{in}}g_{Na}n\left(V_{G}\right)V_{mem}\right]$$

20 $$G=\frac{dx}{dt}=\frac{1}{\tau_{K}}\left[x_{eq}\left(V_{mem}\right)-x\right]$$

Once the two functions *F* and *G*, which define the dynamic equations of the system’s free variables, have been obtained, we can apply realistic parameter values for the transistors in order to analyze the equilibrium curves (nullclines) in the oscillatory region of phase space and the emergence and disappearance of self-sustained oscillations accordingly. To illustrate the different physical behaviors attained by the system, we performed simulations based on the modeling assumptions discussed previously, and the results are shown in Figure 5.

**Figure 5:**
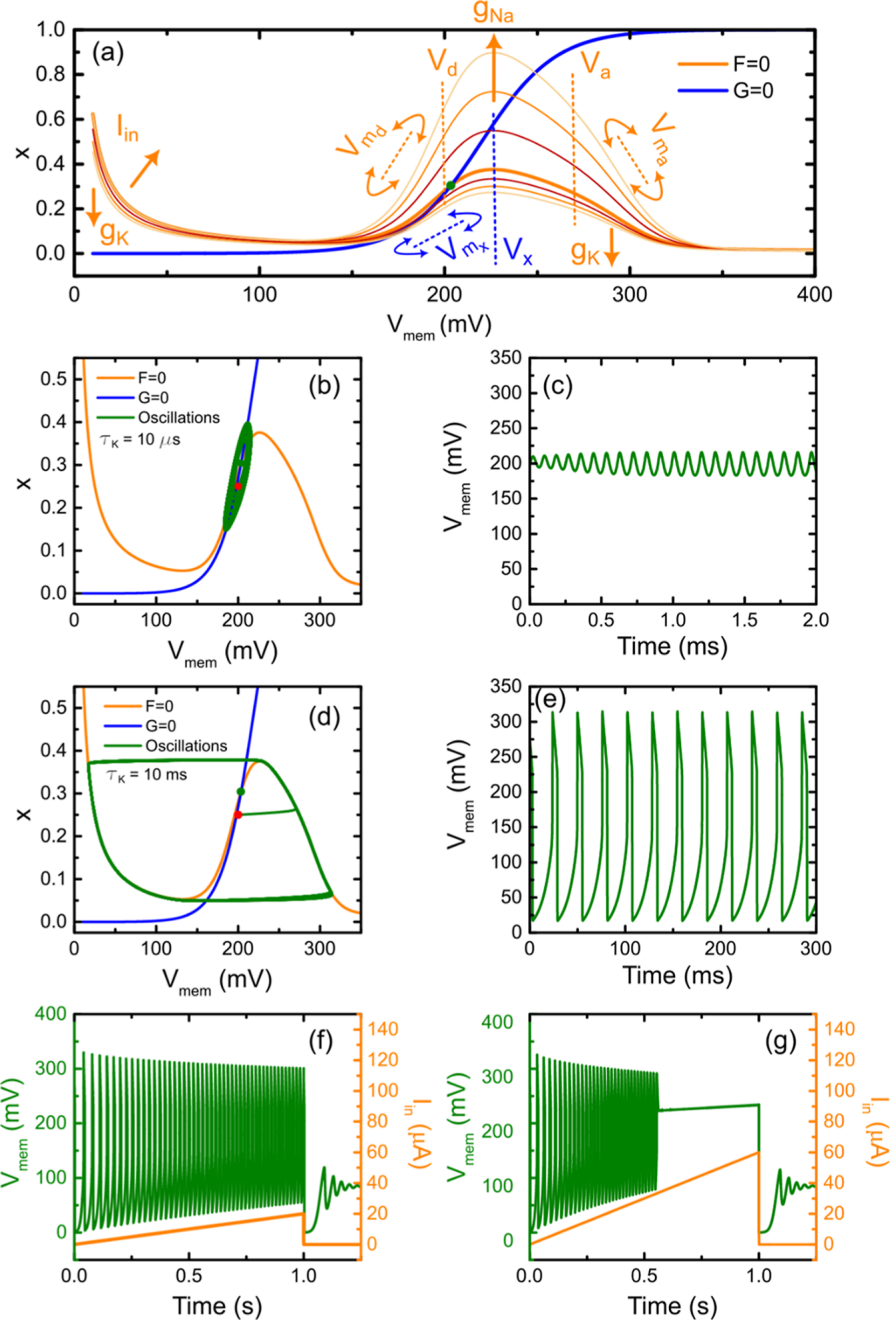
(a) Graphical representation of the nullclines governing the system described in Figures 1a and 2, plotted in the phase space (*x*-*V*_mem_). The thick blue and orange lines correspond to nullclines simulated with the OECTs parameters used in Figure 4 and *C*_m_ = 10*nF*; *I*_in_ = 5μA. The upward-shifted nullclines result from an increase in sodium conductance: *g*_Na_ = 0.15–0.25 mS, while the downward-shifted ones correspond to an increase in potassium conductance: *g*_*K*_ = 0.90–1.10 mS. (b) Nullclines corresponding to the standard parameters and resulting system trajectory (green curve) for a potassium channel relaxation time of τ_*K*_ = 10 μs. The red dot marks the initial condition, and the green dot marks the intersection point of the nullclines. (c) Corresponding voltage time-domain oscillations. (d,e) Same as (b) and (c) but for a potassium relaxation time τ_*K*_ = 10 ms. (f,g) Time-domain evolution of the system under increasing input current (right *y*-axis), illustrating the changes in the oscillatory behavior as this key parameter is varied.

Using the nullclines derived from eqs 3, 4 in 19, 20, we can examine the oscillatory regime of the system. Oscillations occur when the intersection point of the nullclines is inherently unstable. In this configuration, that condition is met when the variable *x* increases with increasing *V*_mem_; that is, when the slope ∂*x*/(∂*V*_mem_) > 0 along the *F* = 0 nullcline. Therefore, if the *G* = 0 nullcline intersects the *F* = 0 nullcline in this region and only once, then a limit cycle oscillation emerges. It should be clarified that although the mentioned slope is positive, the differential resistance is in fact negative because the Na channel is considered to work in a reversed way.

To visualize this analysis, we refer to Figure 5a. Here, we show the shape of the system’s nullclines and identify key parameters that influence their form. Among them, the input current stands out as being the most accessible and impactful. The increase of this current modifies the shape of the *F* = 0 nullcline by increasing its decay at low voltages and narrowing the oscillatory region (i.e., decreasing the region of positive slope in *x* at intermediate voltages). Other parameters, such as the steepness *V*_ma_,*V*_md_,*V*_mx_, threshold voltages *V*_*a*_,*V*_*d*_,*V*_*x*_, and intrinsic conductances *g*_*K*_,*g*_Na_ influence as well the nullclines depending on the experimental performance of the devices. Threshold voltages shift the oscillation range, steepness voltages affect the slope of activation/deactivation, and conductances shift the height of the potassium branch (lowering all of the nullcline) or the sodium activation peak (raising the peak in the nullcline), respectively.

The externally tunable parameters in this system are three fold. First, the relative dynamics between the potassium branch and the membrane capacitor can be adjusted by varying the membrane capacitance, which modifies the characteristic response time of the system. Second, the amplification provided by the inverting amplifier, determined by the ratio *R*_f_/*R*_in_, sets the effective feedback strength and, thus, the operating gain of the circuit. Third, the input current *I*_in_ defines the overall bias level and determines the steady-state working point around which oscillations can develop. All other parameters are fixed by the intrinsic characteristics of the transistors and by the biasing conditions required to reproduce the experimentally observed sodium activation/deactivation window.

Figure 5b and d display typical nullclines with a single intersection located within the oscillatory region, leading to the phase space trajectories shown in the same figures and the corresponding temporal oscillations in Figure 5c and e. The red and green dots indicate the initial and intersection points, respectively. In the first case (b and c), the potassium channel’s relaxation time is short, τ_*K*_ = 10 μs, preventing the system from fully following the fast variable’s nullcline (*F* = 0) and resulting in a compact limit-cycle. In the second case (d and e), a longer relaxation time, τ_*K*_ = 10 ms, allows the system to jump along the fast variable’s nullcline, producing larger amplitude oscillations (bigger limit-cycle) driven by sharper transitions in the negative resistance region, close to relaxation oscillations. This difference in the dynamics produced by the decay time variation is, in fact, similar to what occurs in the saddle-node bifurcation, where the difference in the decay time yields either a typical saddle-node, characterized by more or less compact oscillations, or a saddle-node on invariant cycle that features a large amplitude spike like oscillatory behavior.^40,41^ This strengthens the point of view that apparently different neuromorphic systems are driven in a deeper way by similar differential equations.

Finally, Figure 5f,g explores how varying the input current alters the oscillation regime. In the first case, the current is ramped from 0 to 20 μA over 1 s and then abruptly dropped back to 0 μA. The system exhibits a sudden emergence and smooth decay of oscillations. In the second case, the ramp reaches 60μA. At this higher current, the *F* = 0 null line is sufficiently modified so that the intersection point exits the oscillatory region, suppressing oscillations. When the current decreases, oscillations reappear, again with decreasing amplitude. Notably, increasing the current leads to an increase in oscillation frequency and a reduction in amplitude—consistent with the shrinking of the oscillatory region in the nullcline space.

This qualitative analysis of the nullclines provides a general understanding of how the system’s behavior evolves with changes in the adjustable parameters and how oscillations can be induced or suppressed. However, to translate these insights into practical design criteria and enable the experimental realization of the oscillator, a more quantitative approach is required. In particular, we seek to determine, without resorting to trial and error, the exact parameter ranges that lead to self-sustained oscillations and how the oscillation frequency varies with those parameters.

To this end, we performed a comprehensive parametric bifurcation analysis by computing the trace and determinant of the Jacobian matrix across the relevant parameter space defined by the three externally tunable parameters. For each combination of parameters, trace *T*_λ_ identifies the local stability regime, while determinant Δ_λ_ provides a measure proportional to the oscillation frequency in the unstable region. The resulting bifurcation maps are summarized in Figure 6. In Figure 6a, the diagram is plotted as a function of the input current *I*_in_ and the feedback gain of the inverting amplifier *R*_f_, whereas Figure 6b shows the dependence on *I*_in_ and the characteristic response time of the potassium branch, which can be effectively tuned by adjusting the membrane capacitance.

**Figure 6:**
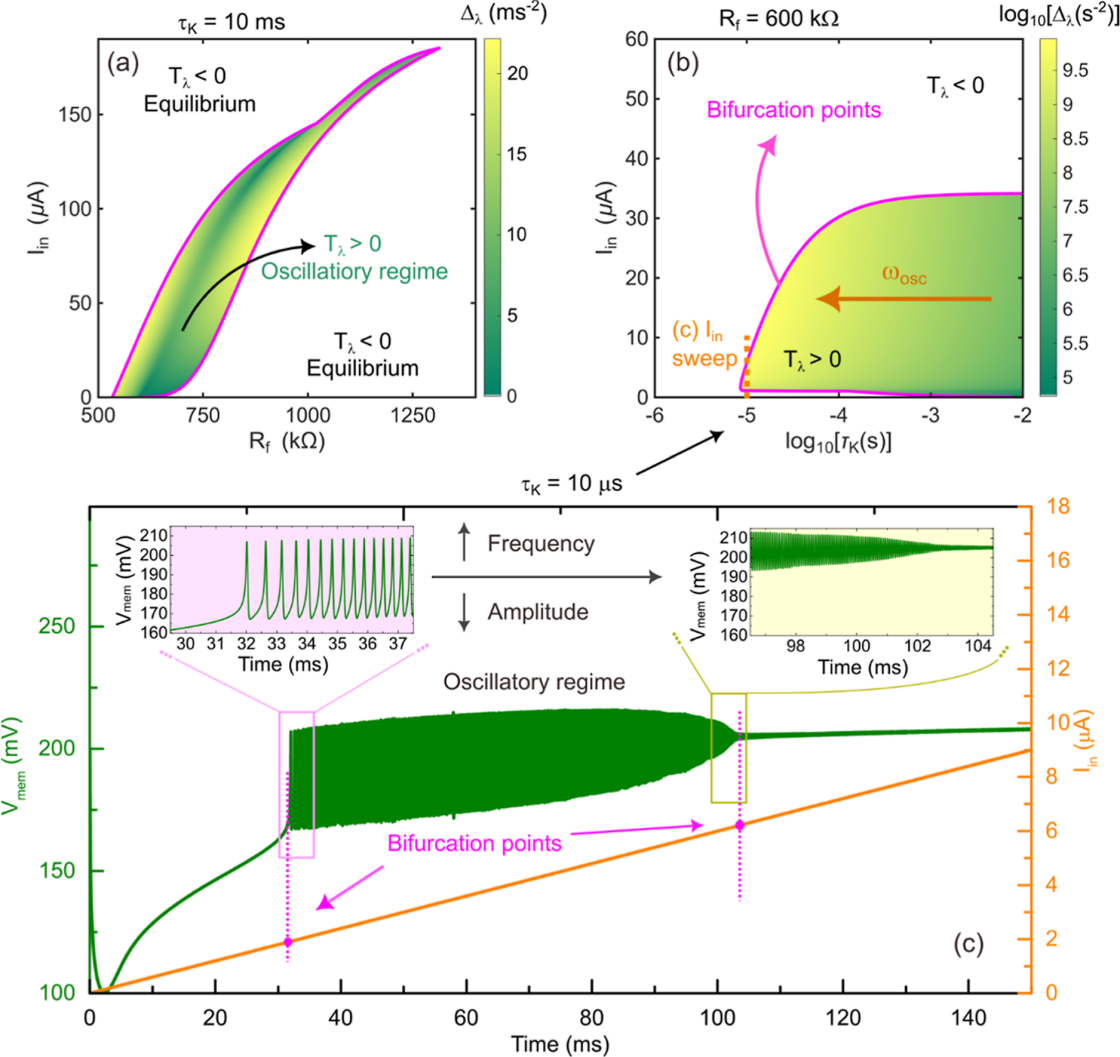
Bifurcation analysis of the different dynamic regimes exhibited by the system. (a,b) Parametric bifurcation diagrams as a function of (a) the input current *I*_in_ and the amplifier gain *R*_f_, and (b) the *I*_in_ and the potassium channel relaxation time τ_*K*_, last one shown on a logarithmic scale. Each diagram maps the stability regimes of the system: white regions correspond to stable equilibria, pink regions denote the bifurcation boundary (sinusoidal oscillations), and green regions indicate unstable equilibria leading to self-sustained oscillations. Within the oscillatory regime, the determinant of the Jacobian, proportional to the oscillation frequency, is encoded in shades of green, with lighter tones representing higher determinant (frequency) values. (c) One-dimensional sweep of the input current *I*_in_ = 60­(μA/*s*)·*t* (solid orange line) corresponding to the dashed orange cut indicated in panel (b), showing the transition between regimes and the variation of oscillation amplitude and frequency as the parameter changes. The parameters used in the simulations are the same as those from Figure 5.

In both maps, the white areas correspond to stable equilibria (*T*_λ_ < 0), the pink contour marks the bifurcation boundary (*T*_λ_ = 0), and the green region denotes the oscillatory regime (*T*_λ_ > 0). Within the oscillatory region, the determinant of the Jacobian is encoded in color intensity, where lighter shades of green correspond to higher determinant values (i.e., higher oscillation frequencies), while darker shades represent lower determinant values (lower frequencies). The color scale used for this mapping is displayed in Figure 6b, and the potassium channel response time is plotted on a logarithmic scale since both quantities vary over several orders of magnitude.

Finally, Figure 6c presents a one-dimensional sweep of the input current parameter for a representative value of the potassium response time, τ_*K*_, illustrating all dynamic regimes of the system. The circuit transitions from a stable steady state to an oscillatory regime via the bifurcation point, and subsequently returns to stability at higher bias values, passing through two critical transitions. Within the oscillatory region, a clear evolution of both frequency and amplitude can be observed, as the input current increases, the oscillation frequency rises substantially, consistent with the determinant trend in the bifurcation diagrams, while the amplitude decreases, reflecting the progressive reduction of the phase-space area enclosed between the nullclines.

All of the previous analysis was performed using a set of two differential equations, corresponding to the two free variables of the system, *x* and *V*_mem_. This simplification was based on the assumption that the activation and deactivation of the sodium channel are instantaneous. To further validate this assumption, we performed a more complete analysis by introducing a third equation that explicitly describes the dynamics of the sodium channel. In this extended model, the system is governed by three coupled equations: the previously defined functions *F* and *G* of eqs 19, 20, together with an additional equation

21 $$H=\frac{dn}{dt}=\frac{1}{\tau_{Na}}\left(n_{eq}-n\right)$$

where *n*_eq_ corresponds to the equilibrium variable given by eqs 15–17, *n* is now treated as an additional dynamic variable, and *τ*_Na_ represents the relaxation time associated with the activation–deactivation dynamics of the sodium channel.

By sweeping τ_Na_ over several orders of magnitude, from nanoseconds to milliseconds, we observe that sustained oscillations persist as long as the sodium relaxation time remains at least one order of magnitude shorter than that of the potassium channel. In this regime, the waveforms and overall dynamics are essentially identical to those obtained under the instantaneous approximation, with only minor variations in amplitude and frequency when τ_Na_ approaches the potassium timescale. However, when both relaxation times become comparable, the oscillations are progressively diminished and eventually disappear. As τ_Na_ becomes faster, these differences vanish completely, confirming that the instantaneous assumption accurately captures the relevant dynamics of the system. The resulting oscillations obtained from the three-variable model for different time-scale ratios are shown in Figure 7.

**Figure 7:**
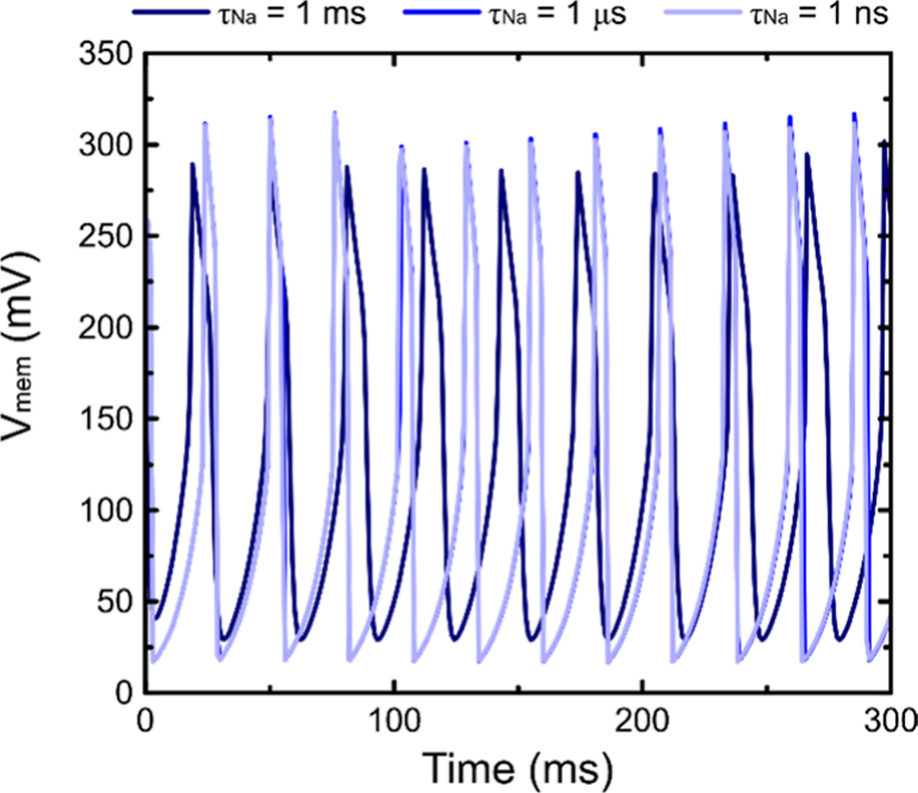
Oscillations obtained from the extended three-variable model, which includes the explicit dynamics of the sodium channel. The plots show the system response for different relaxation times of the sodium channel, nanoseconds, microseconds, and milliseconds, each at least one order of magnitude faster than the potassium channel. The parameters used in the simulations are the same as those from Figure 5.

All these results highlight that in hybrid oscillators, the choice of circuit parameters does not merely define whether oscillations occur but also governs their frequency and amplitude. The interplay among the input current, the amplifier gain, and the membrane dynamics allows the oscillatory behavior to be finely tuned across a wide operational range. Moreover, this tunability enables independent control of oscillation characteristics by adjusting one parameter while keeping others fixed, offering a predictable way to program the dynamic response of the system. Consequently, our analysis demonstrates full control over both the onset and the nature of the oscillations, establishing this circuit analysis as a versatile and customizable platform for neuromorphic and bioelectronic applications.

This oscillator circuit has also been recently applied in other contexts, such as in simple logic circuits that emulate neuronal operations through oscillatory behavior modulated by the mimic of voltage-gated dendritic calcium dynamics.^42^ Furthermore, the negative transconductance exhibited by the OECT has been successfully exploited to develop single-transistor neuron architectures.^7,43^ These advances highlight the critical importance of fully understanding, analyzing, and controlling the various types of responses that these emerging circuits can exhibit.

## 3 Conclusion

In this work, we have developed a simplified yet rigorous dynamic model for analyzing hybrid oscillatory systems. By separating and describing the system in terms of its free variables, we enabled a tractable analysis of a complex circuit that combines classical feedback-based elements such as operational amplifiers with emerging devices exhibiting a negative differential resistance. Despite the apparent complexity of the circuit, this modeling approach allowed us to uncover the fundamental mechanisms responsible for the emergence of oscillations, highlighting the power of dynamic system analysis in capturing the essential behavior with minimal assumptions.

The theoretical framework presented here lays the groundwork for the design and control of a new class of hybrid oscillators. These compact architectures, which blend well-understood feedback mechanisms with novel device-level physics, are capable of producing self-sustained oscillations with high tunability and a minimal component count. The use of phase space analysis, nullcline structure and bifurcation analysis provides not only qualitative insight but also quantitative tools to determine how oscillations arise, how to control their amplitude and frequency, and which device or circuit parameters must be modified to meet specific functional goals. This level of control opens new design possibilities for highly adaptable oscillatory systems.

Altogether, the approach outlined in this paper provides a robust and generalizable foundation for future work on oscillatory architectures, not only in the neuromorphic and bioelectronic domains, but also in broader fields where self-sustained oscillations play a central role, such as in sensing, signal processing, and adaptive control. By grounding oscillator design in a dynamic systems perspective, we enable a new level of predictability and tunability with potential to accelerate the development of next-generation hybrid electronic technologies.

## Data Availability

The code presented here can be accessed at 10.5281/zenodo.16677421 under the license CC BY 4.0 (Creative Commons Attribution 4.0 International).
